# Deep-Learning Approaches for Cervical Cytology Nuclei Segmentation in Whole Slide Images

**DOI:** 10.3390/jimaging11050137

**Published:** 2025-04-29

**Authors:** Andrés Mosquera-Zamudio, Sandra Cancino, Guillermo Cárdenas-Montoya, Juan D. Garcia-Arteaga, Carlos Zambrano-Betancourt, Rafael Parra-Medina

**Affiliations:** 1 Departamento de Patología, Instituto Nacional de Cancerología (INC), Bogotá 111511, Colombia; andres.mosquera@keralty.co (A.M.-Z.); scancino@cancer.gov.co (S.C.); gcardenas@cancer.gov.co (G.C.-M.); 2 Laboratorio de Patología, Clínica Colsanitas, Bogotá 111711, Colombia; 3 Departamento de Ingeniería Eléctrica y Electrónica, Universidad del Norte, Barranquilla 080003, Colombia; 4 Facultad de Ingeniería, Universidad Nacional de Colombia, Bogotá 111321, Colombia; 5 Facultad de Medicina, Universidad Nacional de Colombia, Bogotá 111321, Colombia; judgarciaar@unal.edu.co; 6 Maestría en Estadistica Aplicada y Ciencia de Datos, Universidad El Bosque, Bogotá 111321, Colombia; c.zambrano.betancourt@gmail.com; 7 Instituto de Investigación, Fundación Universitaria de Ciencias de la Salud (FUCS), Bogotá 111411, Colombia

**Keywords:** cancer, computational pathology, cervix, cytology, deep learning, artificial intelligence

## Abstract

Whole-slide imaging (WSI) in cytopathology poses challenges related to segmentation accuracy, computational efficiency, and image acquisition artifacts. This study aims to evaluate the performance of deep-learning models for instance segmentation in cervical cytology, benchmarking them against state-of-the-art methods on both public and institutional datasets. We tested three architectures—U-Net, vision transformer (ViT), and Detectron2—and evaluated their performance on the ISBI 2014 and CNseg datasets using panoptic quality (PQ), dice similarity coefficient (DSC), and intersection over union (IoU). All models were trained on CNseg and tested on an independent institutional dataset. Data preprocessing involved manual annotation using QuPath, patch extraction guided by GeoJSON files, and exclusion of regions containing less than 60% cytologic material. Our models achieved superior segmentation performance on public datasets, reaching up to 98% PQ. Performance decreased on the institutional dataset, likely due to differences in image acquisition and the presence of blurred nuclei. Nevertheless, the models were able to detect blurred nuclei, highlighting their robustness in suboptimal imaging conditions. In conclusion, the proposed models offer an accurate and efficient solution for instance segmentation in cytology WSI. These results support the development of reliable AI-powered tools for digital cytology, with potential applications in automated screening and diagnostic workflows.

## 1. Introduction

Cervical cancer is one of the most commonly diagnosed cancers in women. It is also a significant cause of cancer-related mortality, being the leading cause of death in 36 countries. Cervical cancer remains a critical public health concern, particularly in countries with limited access to preventive measures and early detection [1].

Screening techniques for cervical cancer include the Papanicolaou test (Pap smear), Human papillomavirus (HPV) test, and histopathology. The Pap smear examines cervical cells under a microscope to detect precancerous or cancerous changes, forming the backbone of cervical cancer prevention for decades [2]. The HPV test identifies high-risk HPV types that cause cervical cancer and is often used alone or with the Pap smear (co-testing) to improve detection rates. Histopathology, while not a primary screening tool, confirms diagnoses by analyzing cervical biopsy samples. Together, these methods enable early detection, risk stratification, and timely intervention, reducing the burden of cervical cancer worldwide [2].

The morphological features of the nuclei are critical in the diagnostic interpretations made by pathologists. Key features such as nuclear size, irregularities in the nuclear membrane, and chromatin appearance plays an important role in distinguishing between normal, reactive, and malignant cells. In recent years, advances in molecular biology have deepened our understanding of how genetic and epigenetic factors influence the morphology of the nucleus [3].

A clear example of the significance of nuclear morphology in cytology is its application in cervical cytology, as outlined by the Bethesda system. This system introduced standardized terminology for reporting cervical cytology results, highlighting the importance of nuclear characteristics in identifying cellular abnormalities [4]. These observations show the central role of nuclear morphology in cytological diagnosis and how factors such as HPV infections can affect nuclear structure and diagnostic results.

Moreover, whole slide imaging (WSI) has transformed pathology by providing high-resolution, digitized images that enable detailed morphological and quantitative analyses. This advancement has played a key role in adopting AI-driven methods, enabling large-scale image processing and integration with computational models. The digitization of histopathology slides has also opened the door to computational pathology (cPath), which applies artificial intelligence (AI) for both scientific research and clinical applications [5]. Automation has become essential with the growing workload in pathology laboratories and the global shortage of trained pathologists [6]. By combining digital pathology with computational tools, these technologies offer an efficient and consistent approach to handling high diagnostic volumes, ultimately improving patient care [7].

One prominent application is nucleus segmentation, which involves accurately identifying nuclei within an image by dividing it into distinct regions. Deep learning (DL) has emerged as a transformative tool in this area, with extensive research highlighting its effectiveness and versatility. At its core, image segmentation is the process of dividing a digital image into multiple segments or objects to simplify the analysis and extract valuable information [8].

This study aims to explore the application of advanced computational models in nucleus segmentation in cervical cytology images, focusing on DL methodologies and their comparison with existing approaches reported in the literature.

### Related Work

Automated analysis of cervical images has long been an active area of research, with the first methods dating back to the mid twentieth century [9]. Technological advances in image capture, improved computer vision and machine-learning techniques, and exponential growth of computational power and memory capacity have resulted in constant improvement in both results and expectations, as demonstrated by the multiple surveys and reviews in the area [10,11,12,13].

The appearance of large public anotated image collections allowed, additionally, for different algorithms to be systematically compared on the same data [14].

In the last decade, both Lee et al. [15] and Ushizima et al. [16] initially detected large clumps of cells and then detected “superpixels”, i.e., low-level pixel clusters, which are used for nuclei detection by local thresholding. They then then refined the cytoplasm partition using different graph-based tools.

Lu et al. [17] simultaneously optimized a set of multiple-level set functions in which each function represents a cell within a clump. The function constraints are based on the length of the contour, the strength of the edges, and the shape of the cells as well as on the amount of overlap.

Tareef et al. [18] presented a method which relied on image mathematical morphology and not on machine or deep learning. Specifically, they used multiple passes of the watershed operation of a preprocessed gradient image to refine the cytoplasm and nuclei segmentations. Further improvements are achieved by adjusting contours to the cell shapes.

The appearance of convolutional networks implemented over GPUs shifted research towards simultaneous classification and segmentation of the whole image.

Wan et al. [19] used a deep-learning-based approach by using TernausNet [20] (a modification of U-Net with pretrained encoders) to classify the image pixels into nucleus, cytoplasm, or background and then, a modified DeepLabV2 [21] for semantic segmentation of the cytoplasm.

Assad et al. [22] proposed C-Net, a modification of the U-Net architecture that incorporates cross-scale features in order to learn spatial and local features.

Finally, the transformer deep-learning attention-based model [23] (developed in 2017 initially for text-to-text tasks such as translation) and the vision transformers [24] were rapidly adapted for cervical cell analysis [25].

## 2. Materials and Methods

**Figure 1:**
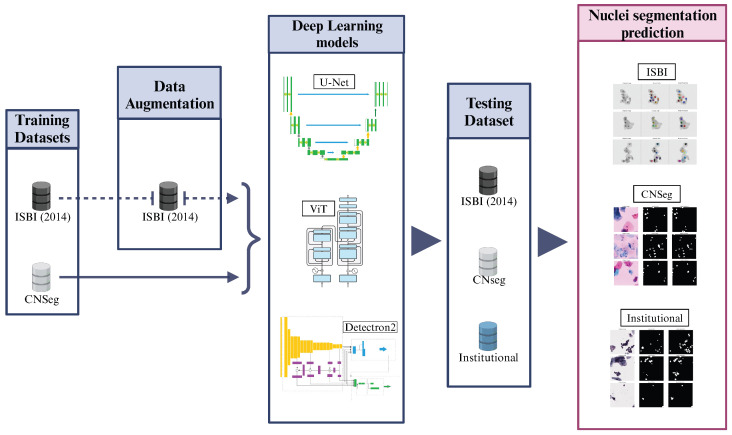
Method pipeline for this study. Architectures adapted from [23,26].

The method pipeline of our study is described in Figure 1. Using public databases, we conducted a study involving different DL methods for nuclei segmentation in liquid-based cervical cytology images. We tested them on our dataset, which consists of WSI cervical cytology images, as described in the following sections. The experiments were performed on Google Colab Pro with a T4 GPU and high RAM capacity. Finally, we compared the performance of the tested DL models with the literature, considering studies that used the same public databases and those that reported performance on their private datasets.

### 2.1. Dataset Description

#### 2.1.1. ISBI 2014 Dataset

The dataset used corresponds to the ISBI 2014 Overlapping Cervical Cytology Image Segmentation Challenge [14], which includes 16 real cervical cytology images with extended depth of field (EDF) and 945 synthetic images with a spatial resolution of 512 × 512 pixels (45 for the training set, 90 for the test set, and 810 for the evaluation set) [17,27]. The images include reference segmentation masks for both the nucleus and cytoplasm. In this work, data augmentation was applied to the ISBI 2014 training set, increasing the number of synthetic images to 3105.

#### 2.1.2. CNSeg Dataset

The CNSeg dataset [28] provided by Zhao et al. [28] was utilized in this research. The dataset is divided into three subsets: the PatchSeg, ClusterSeg, and DomainSeg. In this research, the PatchSeg subset, which includes samples from 200 patients, was used. The Papanicolaou staining technique was used for sample acquisition. For sample digitalization, a Zhiyue scanner (Zhiyu Technology, Xi’an, Shaanxi, China) with a magnification of 40× and a fixed multi-point focusing strategy was used to obtain WSI cervical cell images. These images have a complex background with keratinized cells, microbially infected cells, dark spots, dense glandular cell clusters, atrophic cells, neutrophils, abnormal cell clusters, and cells with unclear boundaries. The WSI images were cropped into patches or images of a spatial resolution of 512 × 512 pixels. Then, 3487 images are randomly selected for the PatchSeg subset, with 3010 images for the training dataset and 477 for the evaluation dataset. Also, every image has cervical cell nuclei boundaries drawn contours as labels. Labeling was a task performed by 20 master’s students trained in nuclei identification and the Labelme software (v5.0.1). After this task, labeled images were checked by two professional pathologists. In total, 85,882 nuclei were labeled in these images.

#### 2.1.3. Institutional Dataset

**Figure 2:**
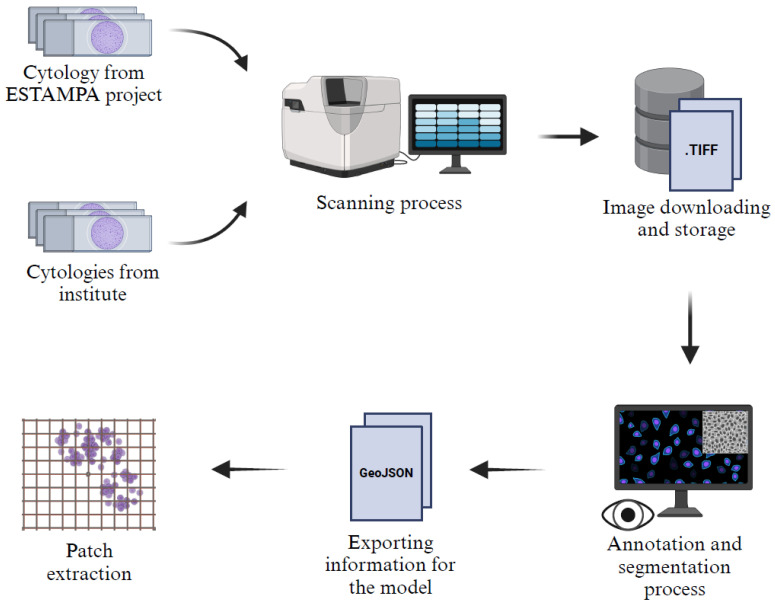
Description of the acquisition and preprocessing pipeline of the institutional dataset.

A description of the acquisition and preprocessing pipeline of the Institutional dataset can be seen in Figure 2. Our dataset consists of 247 patches or images of 512 × 512 pixels, randomly extracted from liquid-based cytology whole slide images WSIs scanned with the VENTANA DP600 slide scanner (Roche Diagnostics, Basel, Switzerland), configured at 40× magnification with three layers and a spacing of 1.0 microns. The images originate from two sources: 48 cytology samples from the ESTAMPA Project [29], reviewed by experts from the International Agency for Research on Cancer (IARC, Lyon, France), and 199 cytology samples collected in an ongoing study at the Instituto Nacional de Cancerología of Colombia (INC), which aims to collect liquid-based cytology samples from women in different regions of Colombia.

All images were annotated using QuPath (v0.5.1) [30] by a master’s student (G. C-M.), trained in the identification of digital cervical nuclei, and a pathologist (A. M-Z.), with expertise in cytology and computational pathology. Nuclei that were considered blurred were excluded from annotation. Once the annotations were completed, the images were processed by extracting patches based on annotation tracking in a GeoJSON format. To optimize the dataset, blank spaces were removed, and only patches containing more than 60% cytologic material were retained, ensuring a more relevant and informative dataset for model training and evaluation.

The datasets generated during the current study are not publicly available but are available from the corresponding author on reasonable request.

### 2.2. Deep-Learning Models

#### 2.2.1. U-Net

Presented by Ronneberger et al., the U-Net model is based on a convolutional neural network (CNN) architecture. U-Net was initially proposed for medical image-segmentation tasks where the datasets usually have a small number of samples. The U-Net principal components consist of an encoder and a decoder. The encoder takes out image features by using convolution and max-pooling layers to downsample the image (contraction process). The decoder upsamples the image and merges it with the corresponding feature maps obtained through the encoder; this process is called expansion [26].

The tested U-Net model architecture consists of 22 encoder and decoder convolutional layers, in addition to maxpooling and dropout layers, with a total of 1,941,105 trainable parameters.

Training configuration had a batch size of 4, a dice loss function, 25 training epochs, and an Adam optimizer with a learning rate of 0.001.

#### 2.2.2. Vision Transformers (ViT)

Vision transformer (ViT) was born from models proposed for natural language processing (NLP) applications. Dosovitskiy et al. [24] demonstrated that ViT was a model suitable for image-classification tasks, and its performance could be better than CNNs if a considerable amount of pretrained data was available. ViT divides the original image into smaller patches, flattens each of them into vectors and uses a transformer encoder to model their relationships.

Tested ViT model was SegFormer-B0, a semantic segmentation pretrained model developed by NVIDIA. It has a total of 5 convolutional layers for encoder and decoder stages. It consists of approximately 3.75 million trainable parameters.

Model training configuration had a batch size of 8. It combined a sigmoid layer and the binary cross-entropy for the loss function, and 50 training epochs were used. An Adam optimizer with a learning rate of 0.0001 was selected.

#### 2.2.3. Detectron

Detectron, proposed by Wu et al., is a modular and optimized deep-learning framework developed by Facebook AI [31] for instance-segmentation tasks. Its approach consists of two principal steps: first, a region proposal network (RPN) identifies candidate regions that are likely to contain objects to be segmented. Then, a Mask R-CNN classifier assigns each region to a specific object category and generates the corresponding segmentation mask. In this study, we implemented an instance-segmentation model using Detectron2, leveraging its Mask R-CNN architecture, which extends Faster R-CNN by integrating a segmentation branch for pixel-wise nuclei mask generation.

The tested detectron model consists of a Mask R-CNN model with a ResNet-50 backbone and a feature pyramid network (FPN) used for instance-segmentation tasks. It has a total of 65 convolutional layers for the ResNet-50 backbone, the FPN, the region proposal network (RPN), and the ROI mask head. It consists of approximately 44 million trainable parameters.

The model training configuration had a batch size of 2. A combination of loss functions as cross-entropy, binary cross-entropy and others were used in different model stages. It used stochastic gradient descent optimizer with a learning rate of 0.00025. The number of training epochs was 1000.

### 2.3. Evaluation Metrics

For model performance in the image-segmentation task, three evaluation metrics are used in this work: Sørensen–dice coefficient (DSC), Jaccard index, or intersection over union (IoU) and PQ.

#### 2.3.1. Sørensen–Dice Coefficient (DSC)

DSC is a statistical measure commonly used as an image-segmentation evaluation metric. It estimates the overlapping between two regions, which in image processing is equivalent to evaluating similarity between a predicted segmentation and a ground truth segmentation. Similarity is quantified as 1 for complete overlapping between regions and 0 for no overlapping. For the DSC mathematical equation, see Equation (1) below.

(1) $$DSC=\frac{2|A\cap B|}{\left|A|+|B\right|}$$

where *A* is the group of pixels of the predicted segmentation region, *B* is the group of pixels of the ground truth segmentation region and |A∩B| is the number of overlapping pixels between *A* and *B*.

DSC is selected in this research as one of the performance metrics because it is broadly used in medical-image segmentation evaluation, and because of the robustness of the overlapping estimation, regardless of the size of segmented regions [32].

#### 2.3.2. Jaccard Index or Intersection over Union (IoU)

IoU is another statistical measure commonly used in image segmentation. It also estimates the overlapping or similarity between predicted segmentation and ground truth segmentation regions, changing some of the mathematical set operations used in DSC. Also, the similarity ranges between 0 for no overlapping and 1 for complete region overlapping. The IoU equation can be seen in Equation (2) below.

(2) $$IoU=\frac{\left|A\cap B\right|}{\left|A\cup B\right|}$$

where *A* is the group of pixels of the predicted segmentation region, *B* is the group of pixels of the ground truth segmentation region, |A∩B| is the number of overlapping pixels between A and B, and |A∪B| is the total number of pixels of region A and region B.

The principal differences between DSC and IoU are that the latter can be more robust to imbalanced classes. Moreover, IoU penalizes stronger than DSC under-segmentation and over-segmentation [32,33].

#### 2.3.3. Panoptic Quality (PQ)

PQ can evaluate semantic segmentation or pixel assignment to each class, in addition to instance segmentation or object instances assignments to each class. Also, PQ calculation is divided into two elements: recognition quality (RQ) or the well-known F1 score related to the accuracy of object identification, and semantic quality (SQ) or the average IoU, which indicates the accuracy of segmentation for correctly matched regions. These characteristics make PQ a more complete evaluation metric. For the PQ, RQ, and SQ mathematical equations, see Equations (3) and (4) below.

(3) $$PQ=\frac{\sum_{(p,g)\in TP}IoU\left(p,g\right)}{\left|TP|+0.5|FP|+0.5|FN\right|}$$

(4) $$\begin{array}{cc} RQ & =\frac{\left|TP\right|}{\left|TP|+0.5|FP|+0.5|FN\right|}\\ SQ & =\frac{\sum_{(p,g)\in TP}IoU\left(p,g\right)}{\left|TP\right|} \end{array}$$

where TP are true positives, FP are false positives, and FN are false negatives. In addition, $\frac{\sum_{(p,g)\in TP}IoU\left(p,g\right)}{\left|TP\right|}$ is the average IoU of matched regions, and 0.5|FP|+0.5|FN| in the denominator, is used to penalize no-matched regions.

As PQ is estimated for each class independently and then through an average over classes, PQ is more robust to class imbalance. On the other hand, PQ evaluation depends on an IoU threshold, it could artificially decrease the performance score [34].

### 2.4. Image Acquisition and Z-Stacking

Whole-slide images (WSIs) from the institutional dataset were acquired using the VENTANA DP600 scanner (Roche Diagnostics, Basel, Switzerland) configured with three focal planes (−1, 0, +1 µm from the central plane) at 40× magnification. This protocol specifically addressed the focal variability characteristic of circle smear preparations in liquid-based cytology, where cellular overlapping.

The proprietary VENTANA software (v1.0) automatically merged the Z-stack layers, eliminating manual processing while preserving optimal focus areas. This process generated standardized digital files of 5–7 GB per WSI, with the exclusion of high cellular density areas where the −1/+1 planes failed to achieve complete focus and low cellularity zones lacking adequate focal references.

## 3. Results

A total of 4679 digital cervical cytology images or patches were analyzed for nuclei segmentation. The performance evaluation of the tested models—U-Net, vision transformer (ViT), and detectron—was conducted on three different datasets: ISBI 2014, CNseg, and an evaluation set consisting of our dataset or institutional dataset, where models were trained exclusively on CNseg. The evaluation metrics used include the dice similarity coefficient (DSC), intersection over union (IoU), and panoptic quality (PQ), providing a comprehensive assessment of segmentation accuracy. Results are shown in Table 1.

### 3.1. Performance of DL Models on the ISBI 2014 Dataset

In the ISBI 2014 dataset, detectron and ViT achieved the highest DSC score (0.89), outperforming U-Net (0.85). The IoU metric also showed a superior performance for detectron (0.82), slightly higher than ViT (0.81). However, detectron significantly outperformed all models in PQ (0.98), indicating its strong instance segmentation capability. These results suggest that detectron is particularly effective at segmenting individual objects while maintaining a high overlap with ground-truth annotations.

**Figure 3:**
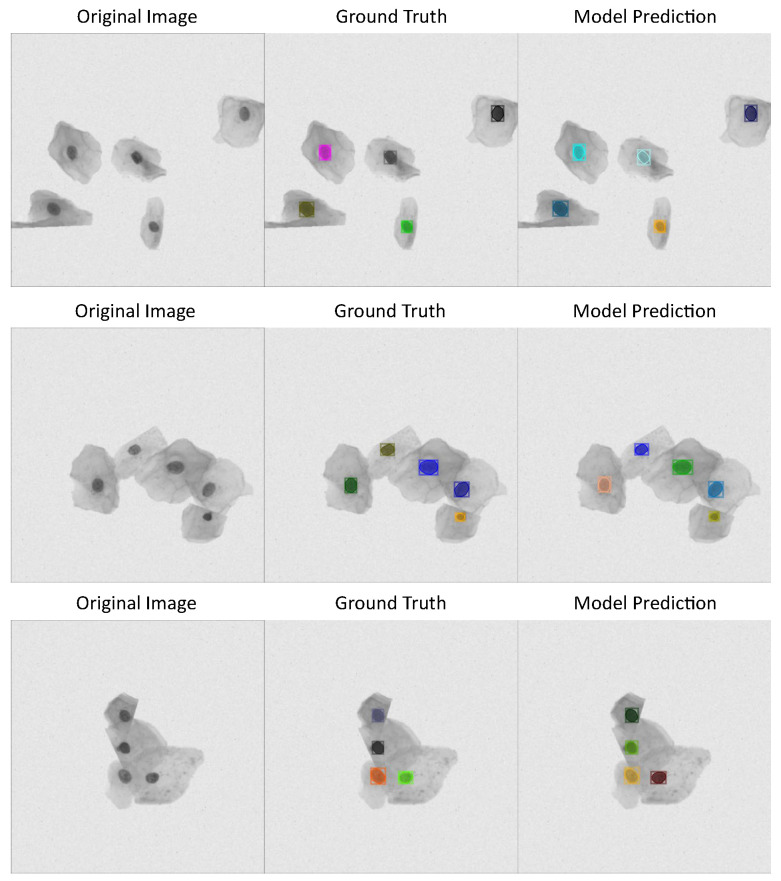
Detectron model results using ISBI 2014 dataset: first column shows cervical cytology images, second column shows the annotation or images ground truth, last column shows the predictions made by the model.

See Figure 3 with three different cervical cytology images from ISBI 2014 dataset, their corresponding annotations, and the predictions made by detectron model, which has the best performance metrics according to Table 1.

### 3.2. Performance of DL Models on the CNseg Dataset

When evaluating models on the CNseg dataset, U-Net demonstrated the best performance across all metrics (DSC = 0.86, IoU = 0.75, PQ = 0.86). In contrast, ViT and detectron showed slightly lower IoU values (0.72 each), with detectron achieving a slightly higher PQ (0.84) than ViT (0.77). The results indicate that U-Net retains strong segmentation capabilities for this dataset, likely due to its efficient feature extraction and spatial preservation.

**Figure 4:**
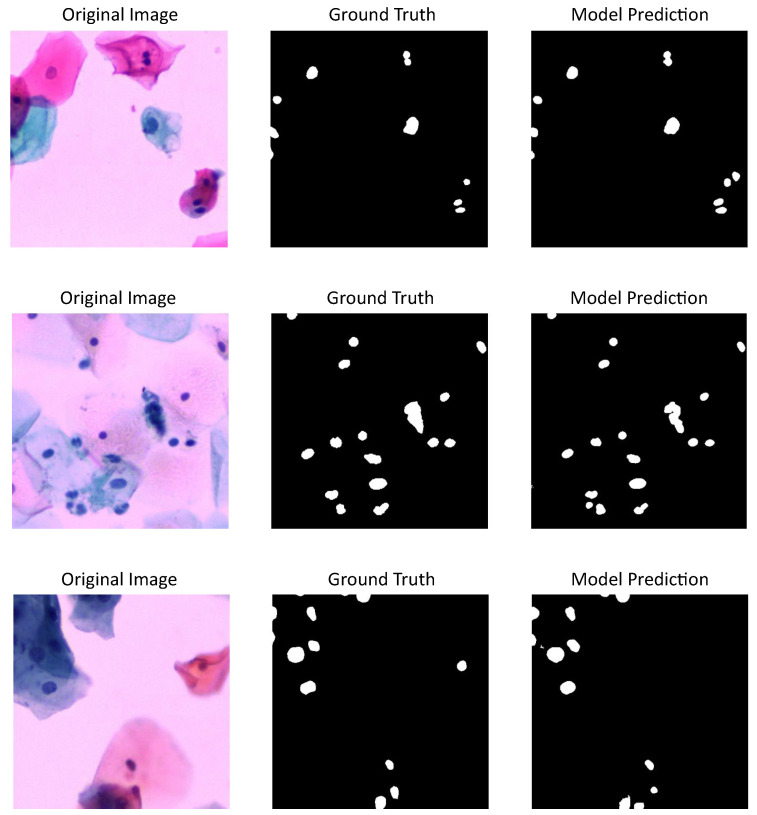
U-Net model results using CNSeg dataset: first column shows cervical cytology images, second column shows the annotation or images ground truth, last column shows the predictions made by the model.

Figure 4 shows three cervical cytology images from CNseg dataset, their nuclei annotations, and the predictions made by U-Net model, which has the highest performance metrics according to Table 1.

### 3.3. Performance of DL Models on Our Dataset

When models trained on CNseg were tested on our dataset, U-Net remained the best-performing model with a DSC of 0.62, IoU of 0.45, and PQ of 0.62. ViT exhibited a similar IoU (0.45) but a significantly lower PQ (0.25), suggesting challenges in accurate instance-level segmentation. Detectron showed the lowest overall performance (DSC = 0.51, IoU = 0.38, PQ = 0.31), indicating a reduced ability to generalize to an unseen dataset.

These results highlight the impact of the change in capture conditions, e.g., staining procedures, microscope quality, personnel experience, etc. In our case, this refers to the fact that although most cells were sharply captured, some regions—particularly those with three-dimensional cell clusters—appeared blurred due to scanning artifacts. The staining was performed using the ThinPrep technique, and we only annotated cells with clearly visible nuclei. Cells that were not entirely in focus were excluded from the annotations.

**Figure 5:**
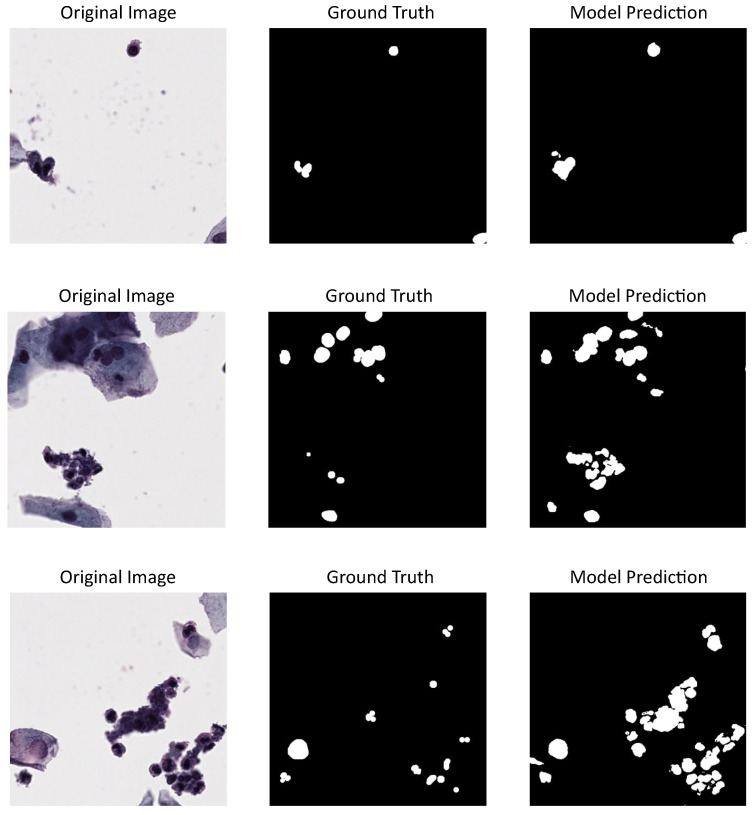
U-Net model results using our dataset for evaluation and CNSeg for training: first column shows cervical cytology images, second column shows the annotation or images ground truth, last column shows the predictions made by the model.

Figure 5 shows three different cervical cytology images from our dataset, their annotations or ground truth, and the predictions made by U-Net model, which proved to be the best-tested model. Also, a nuclei over-segmentation can be seen coming from the DL model predictions compared to the annotations, which decreases the performance metrics shown in Table 1. This could be happening because of the excluded annotations in our dataset.

In addition, tested models’ evaluation in our dataset should not only include segmentation accuracy but also computational efficiency, which is very important for clinical implementation. For this purpose, we measured execution time and other computational resources for the prediction task of nuclei segmentation using the evaluation set (247 patches from the institutional dataset). Results are shown in Table 2.

Even though metrics for models trained on CNseg and tested on our dataset showed the highest segmentation performance for the U-Net model, measured computational resources show a shorter execution time than the ViT model and less RAM usage than the detectron model.

## 4. Discussion

DL models have been widely used for nuclei-segmentation tasks in cervical cytology images. In this work, three different deep-learning approaches have been selected to evaluate their performance on the public datasets described above and also with our dataset. Previous work has shown that models such as U-Net, vision transformers, and Detectron2 yield good results in object segmentation across various types of images, including medical images [35].

Table 3 presents a comparative analysis of our tested DL models against the methods introduced by Zhao et al. (2023) [28] using the ISBI 2014 dataset, with PQ as the primary evaluation metric. While the approaches by Zhao et al. [28] achieved an average PQ of 0.72 ± 0.02 across seven models, our three tested models attained a significantly higher PQ of 0.88 ± 0.09, representing a 22.2% improvement. This increase suggests that the tested methods provide more precise segmentation and instance recognition, probably because of advancements in model architecture selection and training strategies. Despite achieving superior results, our models exhibited slightly higher performance variability (±0.09 vs. ±0.02), indicating room for further optimization.

Table 4 shows the tested models’ performance against existing approaches using DSC metric on the ISBI 2014 dataset. The highest DSC was obtained by Rasheed et al. (2023) [22] with 0.94 ± 0.04, followed closely by Wan et al. (2019) [19] with 0.93 ± 0.04. Our models achieved a DSC of 0.88 ± 0.02, which is competitive with other state-of-the-art approaches, such as Tareef et al. (2018) [18] with 0.89 ± 0.07 and Lee et al. (2016) [15] with 0.90 ± 0.08.

Although our models did not achieve the highest DSC score, they demonstrated greater stability, as indicated by the lowest standard deviation (±0.02) among all compared methods. This suggests that while certain individual approaches may achieve marginally higher DSC values, the tested methods ensure more consistent and robust segmentation performance across different cases.

Table 5 provides a comparative analysis of the tested deep-learning models in relation to the methods introduced by Zhao et al. (2023) [28], using the CNseg dataset and evaluating performance based on PQ. While Zhao et al.’s [28] 11 models achieved an average PQ of 0.66 ± 0.05, our three proposed models significantly outperformed them with a PQ of 0.82 ± 0.05, representing a 24.2% improvement. Despite using fewer models, our approach demonstrates superior segmentation performance, likely due to model architecture selection and optimized training strategies.

**Figure 6:**
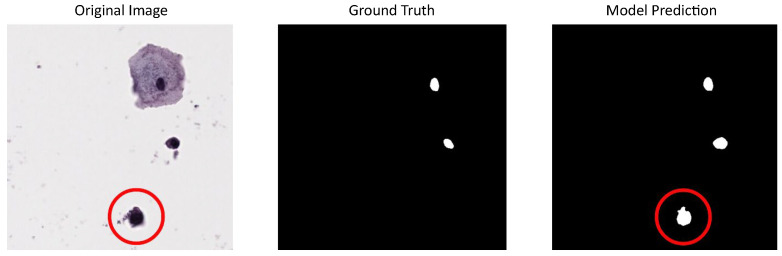
U-Net model result using our dataset for evaluation and CNSeg for training: first column shows a cervical cytology image with a blurred nucleus circled in red, second column shows the corresponding annotation (the blurred nucleus is excluded), last column shows the predictions made by the model that includes the blurred nucleus.

Due to the limited number of images available in our dataset for model training but the sufficient number for evaluation, we used CNseg for training and reserved our dataset for testing. This choice was made because CNseg cytology images exhibit characteristics similar to those of our dataset, allowing the models to learn relevant features while ensuring a robust evaluation on an independent set. Performance metrics for the tested models using our dataset (or institutional dataset) are summarized in Table 1. It can be seen that these metrics are lower than when using public datasets, similar to results obtained in other works that also use private datasets like the one in Hu et al. (2024) [36], where the DSC of the best-proposed model is 0.68 and the IoU is 0.51, values that are close to the results using our dataset. Particular characteristics in the image acquisition and digitalization process for private datasets could lead to this performance decreasing. In our dataset (institutional dataset), in the random mode, some image regions contained cells with blurred nuclei, making annotation more challenging and less reliable compared to clearly defined nuclei. Despite this, the best-tested model was able to detect even these blurred nuclei, which could potentially support future models in the generation of synthetic images. An example of this situation can be seen in Figure 6.

Although our dataset was generated using the Z-stacking method, we observed that some areas appeared blurred upon downloading the images, despite not being noticeable when viewed in the scanner’s native platform. This issue could be attributed to the inherent limitations of Z-stacking, such as the inclusion of multiple focal planes leading to increased file sizes and potential misalignment of focus layers. Additionally, the variability in cellular distribution across cytology slides, particularly in smeared or liquid-based preparations, may contribute to these inconsistencies. We consider that volumetric scanning could contribute significantly to overcoming the limitations associated with flat digital representations of cytology slides. By integrating multiple focal planes into a single composite image that selectively retains only the most in-focus regions, this approach enhances the visualization of three-dimensional cell clusters and overlapping structures commonly found in cytological preparations. In addition to improving image sharpness, volumetric scanning reduces redundant pixel information generated through Z-stacks, leading to more efficient storage and faster image rendering. These benefits are particularly relevant in computational pathology workflows, where high-quality input data directly impacts the performance of downstream tasks such as cell segmentation and feature extraction. Moreover, compression algorithms optimized for multi-plane acquisition can further reduce file sizes without compromising diagnostically relevant information, making volumetric scanning a promising strategy for large-scale cytology digitization [37,38].

Regarding computational resources required for tested models when Institutional dataset patches were used as the evaluation dataset, we can see that in general, execution time was very good: the worst cases is less than a minute for nuclei segmentation of 247 patches (see Table 2). This means that for clinical implementation, the tested models would be a fast and useful tool for cell analysis in pathological applications. Specifically, from the three tested models, the ViT model had the shortest execution time; however, it also required the biggest RAM usage capacity. This is a trade-off for that solution, and its implementation depends on the available computational capacity. Detectron would be a suitable solution with little RAM usage requirements, but performance metrics for nuclei segmentation were the lowest from the tested models (see Table 1). U-Net was the slowest approach regarding the prediction task for nuclei segmentation with a considerable amount of RAM usage, but it had the best performance metrics.

## 5. Conclusions

Our study demonstrates that the tested DL models achieve superior segmentation performance compared to existing approaches. When evaluated on public datasets, our models consistently outperformed previously reported methods, showing improved segmentation accuracy and instance recognition. The combination of optimized architectures and robust training strategies contributed to this outcome.

Despite strong performance on public datasets, we observed a performance drop when evaluating on our institutional dataset. However, we consider that the observed over-segmentation highlights the ability of tested models to detect nuclei that, although not identified in the annotation, were correctly recognized by the model. This suggests the potential of the mentioned models to support synthetic image generation, thereby improving segmentation in cases with poor image quality. We also observed that with the available computational resources, tested models had a very good execution time for nuclei segmentation of the whole evaluation dataset (247 patches of the institutional dataset), meaning that they could be a good approach for clinical implementation.

Our findings emphasize the limitations of Z-stacking in cytology imaging, as some regions appeared blurred upon image download despite being clear in the scanner’s native platform. We propose that volumetric scanning could address these challenges by optimizing focus selection, reducing redundant data, and enhancing the efficiency of digital cytology processing.

## Figures and Tables

**Table 1:** Comparison of tested models across different datasets.

Dataset	Tested Models	DSC	IoU	PQ
ISBI 2014	U-Net	0.85	0.75	0.86
	ViT	**0.89**	0.81	0.81
	Detectron	**0.89**	**0.82**	**0.98**
CNseg	U-Net	**0.86**	**0.75**	**0.86**
	ViT	0.84	0.72	0.77
	Detectron	0.80	0.72	0.84
Institutional dataset *	U-Net	**0.62**	**0.45**	**0.62**
	ViT	0.61	**0.45**	0.25
	Detectron	0.51	0.38	0.31

* This dataset was used exclusively for evaluation, utilizing the prior training performed with the CNseg database. **Bold numbers** are used for the highest values of performance metrics.

**Table 2:** Measured computational resources for the nuclei segmentation of tested models on the evaluation set.

Tested Models	Execution Time (s)	RAM Usage (GB)	GPU Type
U-Net	48.12	29.28	NVIDIA T4
ViT	**3.53**	47.29	NVIDIA T4
Detectron	25.13	**5.53**	NVIDIA T4

**Bold numbers** are used for the lowest values of performance metrics.

**Table 3:** A comparison with other approaches using ISBI 2014 Dataset and PQ performance metric.

Methods	Number of Methods	PQ
Zhao et al. (2023) proposed methods [28]	7	0.72±0.02
Tested methods	3	0.88±0.09 *

* Average PQ performance using our three tested DL models. **Bold numbers** are used for the highest values of performance metrics.

**Table 4:** A comparison with other approaches using ISBI 2014 Dataset and DSC performance metric.

Methods	Number of Methods	DSC
Rasheed et al. (2023) proposed methods [22]	1	0.94±0.04
Tareef et al. (2018) proposed methods [18]	1	0.89±0.07
Lu et al. (2017) proposed methods [27]	3	0.87±0.08
Lee et al. (2016) proposed methods [15]	1	0.90±0.08
Lu et al. (2015) proposed methods [17]	1	0.88
Wan et al. (2019) proposed methods [19]	1	0.93±0.04
Tested methods	3	0.88±0.02

**Bold numbers** are used for the highest values of performance metrics.

**Table 5:** A comparison with other approaches using CNseg Dataset and PQ performance metric.

Methods	Number of Methods	PQ
Zhao et al. (2023) proposed methods [28]	11	0.66±0.05
Tested methods	3	0.82±0.05

**Bold numbers** are used for the highest values of performance metrics.

## Data Availability

The datasets are available in their original repository. For CNSeg: https://www.kaggle.com/datasets/zhaojing0522/cervical-nucleus-segmentation, accessed on 30 March 2025 and ISBI 2014: https://cs.adelaide.edu.au/~carneiro/isbi14_challenge/dataset.html, accessed on 30 March 2025.
